# Transmission characteristics of MERS and SARS in the healthcare setting: a comparative study

**DOI:** 10.1186/s12916-015-0450-0

**Published:** 2015-09-03

**Authors:** Gerardo Chowell, Fatima Abdirizak, Sunmi Lee, Jonggul Lee, Eunok Jung, Hiroshi Nishiura, Cécile Viboud

**Affiliations:** School of Public Health, Georgia State University, Atlanta, Georgia USA; Division of Epidemiology and Population Studies, Fogarty International Center, National Institutes of Health, Bethesda, Maryland USA; Department of Applied Mathematics, Kyung Hee University, Yongin-si, 446-701 Republic of Korea; Department of Mathematics, Konkuk University, 120 Neungdong-ro, Gwngjin-gu, Seoul, 143-701 Republic of Korea; Graduate School of Medicine, The University of Tokyo, Tokyo, Japan; CREST, Japan Science and Technology Agency, Saitama, Japan

**Keywords:** Coronavirus, Exposure pattern, Hospital transmission, MERS, Middle East, Nosocomial, Reproduction number, SARS, South Korea

## Abstract

**Background:**

The Middle East respiratory syndrome (MERS) coronavirus has caused recurrent outbreaks in the Arabian Peninsula since 2012. Although MERS has low overall human-to-human transmission potential, there is occasional amplification in the healthcare setting, a pattern reminiscent of the dynamics of the severe acute respiratory syndrome (SARS) outbreaks in 2003. Here we provide a head-to-head comparison of exposure patterns and transmission dynamics of large hospital clusters of MERS and SARS, including the most recent South Korean outbreak of MERS in 2015.

**Methods:**

To assess the unexpected nature of the recent South Korean nosocomial outbreak of MERS and estimate the probability of future large hospital clusters, we compared exposure and transmission patterns for previously reported hospital clusters of MERS and SARS, based on individual-level data and transmission tree information. We carried out simulations of nosocomial outbreaks of MERS and SARS using branching process models rooted in transmission tree data, and inferred the probability and characteristics of large outbreaks.

**Results:**

A significant fraction of MERS cases were linked to the healthcare setting, ranging from 43.5 % for the nosocomial outbreak in Jeddah, Saudi Arabia, in 2014 to 100 % for both the outbreak in Al-Hasa, Saudi Arabia, in 2013 and the outbreak in South Korea in 2015. Both MERS and SARS nosocomial outbreaks are characterized by early nosocomial super-spreading events, with the reproduction number dropping below 1 within three to five disease generations. There was a systematic difference in the exposure patterns of MERS and SARS: a majority of MERS cases occurred among patients who sought care in the same facilities as the index case, whereas there was a greater concentration of SARS cases among healthcare workers throughout the outbreak. Exposure patterns differed slightly by disease generation, however, especially for SARS. Moreover, the distributions of secondary cases per single primary case varied highly across individual hospital outbreaks (Kruskal–Wallis test; *P* < 0.0001), with significantly higher transmission heterogeneity in the distribution of secondary cases for MERS than SARS. Simulations indicate a 2-fold higher probability of occurrence of large outbreaks (>100 cases) for SARS than MERS (2 % versus 1 %); however, owing to higher transmission heterogeneity, the largest outbreaks of MERS are characterized by sharper incidence peaks. The probability of occurrence of MERS outbreaks larger than the South Korean cluster (n = 186) is of the order of 1 %.

**Conclusions:**

Our study suggests that the South Korean outbreak followed a similar progression to previously described hospital clusters involving coronaviruses, with early super-spreading events generating a disproportionately large number of secondary infections, and the transmission potential diminishing greatly in subsequent generations. Differences in relative exposure patterns and transmission heterogeneity of MERS and SARS could point to changes in hospital practices since 2003 or differences in transmission mechanisms of these coronaviruses.

**Electronic supplementary material:**

The online version of this article (doi:10.1186/s12916-015-0450-0) contains supplementary material, which is available to authorized users.

## Background

The Middle East respiratory syndrome coronavirus (MERS) is a zoonotic pathogen that has caused recurrent spillovers in the human population since March 2012 [1]. A total of 1,047 laboratory-confirmed cases of infection with MERS including 460 deaths have been reported in Saudi Arabia alone as of 15 July 2015 [2]; the concentration of human infections in this region is thought to be linked to the local population of dromedary camels, which may serve as an intermediate host for MERS [3, 4]. The human-to-human transmission potential of MERS is thought to be subcritical [1, 5, 6], although there is occasional amplification in the healthcare setting [5, 7–11]. While sporadic importations of MERS to Europe, Africa, Asia, and North America via returning travelers from the Middle East had not sparked local outbreaks until recently, a single importation into South Korea on 4 May 2015 triggered the largest cluster of cases outside the Middle East to date [12]. The index patient was a 68-year-old businessman who visited several countries in the Middle East before returning to South Korea via Qatar [13, 14], where he developed respiratory symptoms on 11 May 2015. An accurate diagnosis of MERS was not established until 20 May 2015, after the index patient had sought treatment in several different healthcare facilities [13]. A total of 186 MERS infections in South Korea have been linked to the healthcare facilities visited by the index patient and subsequent infections. As a result of this large cluster, more than 6,000 contacts have been monitored in South Korea [13–15].

Although large-scale community transmission has not been reported for MERS, large hospital clusters are not infrequent and can amplify transmission, which aligns with the transmission characteristics of severe acute respiratory syndrome (SARS), a related coronavirus that sparked global concern in 2002–2003 [10, 11, 16, 17]. Coronaviruses associated with both syndromes have high affinity to the lower respiratory tract and cause severe pneumonia [18–20], particularly among older adults with underlying medical conditions [21, 22]. Both viruses are thought to be associated with some degree of transmission heterogeneity, indicating that super-spreading events are expected [23, 24].

While the individual heterogeneity of MERS (i.e. variation in the transmissibility by individuals) has been explored recently [25, 26], here we focus exclusively on hospital outbreaks, where transmission is amplified. Further, we provide the first head-to-head comparison with SARS and carry out a comparative analysis of the transmission characteristics and exposure patterns of previously reported hospital clusters of MERS and SARS to assess the unexpected nature of the recent South Korean nosocomial outbreak and estimate the probability of future large hospital clusters.

## Methods

We analyzed a variety of epidemiological datasets to quantify the exposure patterns and transmission characteristics of MERS and SARS by disease generation and in different settings, including aggregated case counts, individual-level case data, and detailed transmission trees, as detailed below.

### Individual-level case data to quantify exposure patterns

#### Middle East respiratory syndrome

We analyzed a publicly available line list of MERS cases reported between March 2013 and May 2015 to the Kingdom of Saudi Arabia (KSA) Ministry of Health [2]. For each case, we obtained the date of reporting, healthcare worker status, and whether the infection had been linked to healthcare facilities. Healthcare facilities in KSA report MERS cases to the Ministry of Health through an electronic case-reporting system once all appropriate testing is complete [27]. Case confirmation is based on laboratory diagnosis through detection of viral nucleic acid or serology, regardless of the presence of clinical signs and symptoms [28].

#### Severe respiratory syndrome

Total SARS case counts for Canada, China, Hong Kong, Singapore, and Vietnam including cases among healthcare workers were obtained from the World Health Organization (WHO) website for the outbreaks in 2003 [29]. A probable case of SARS was defined as radiographic evidence of pneumonia or respiratory distress syndrome on a chest X-ray, positivity for SARS virus infection by one or more laboratory assays, or autopsy findings consistent with the pathology of respiratory distress syndrome [30]. A confirmed case was defined based on a positive laboratory test combined with clinical evidence compatible with SARS.

### Transmission trees to quantify transmission characteristics by generation time

We obtained and analyzed detailed transmission trees for hospital clusters of MERS and SARS. Transmission trees provide information on the epidemiological links between successive cases and allow for quantification of the reproduction number, R, which is a key parameter in outbreak investigations. In general, R quantifies the transmission potential of an infectious pathogen, which informs the likelihood of large-scale outbreaks [31, 32]. Estimates of R > 1 indicate the potential for an infectious pathogen to generate a major outbreak while R < 1 indicates that transmission of a given pathogen cannot be sustained in the population. Here, we note R_g_ as the reproduction number at disease generation g, where g ≥ 0. If g = 0 then R_0_ denotes the index reproduction number, or the number of secondary cases ascribed to the index case in a given outbreak. Overall, the reproduction number is a function of several inter-related factors, including the epidemiology of the disease, local cultural factors, and environmental conditions. Moreover, the reproduction number is affected by population behavior changes and control interventions occurring over the course of an epidemic.

We defined hospital clusters as outbreaks that started in a healthcare setting with a hospitalized patient (the index case) and ended when the chain of transmission subsided and no further infections were linked to the healthcare setting. We searched for past MERS and SARS outbreaks with information on types of exposure. Specifically, we searched PubMed for articles on SARS published after 1 January 2003 with the search “(SARS AND hospital) OR (SARS AND healthcare)” and articles on MERS published after January 2012 with the search “(MERS AND hospital) OR (MERS AND healthcare).” We also screened relevant articles cited within selected articles.

Overall, we found information on types of exposure for nine hospital outbreaks (three MERS and six SARS), while detailed transmission trees were available for four of these (two SARS and two MERS clusters) [11, 13, 14, 16, 17, 33, 34]; an ill-defined SARS transmission tree from Taiwan had to be discarded.

Each case within the hospital cluster was classified according to occupational and social exposure, including healthcare workers, patients, family members and visitors, and non-clinical staff. Healthcare workers were defined as personnel responsible for the direct care of patients and included physicians, nurses, laboratory technicians, and emergency medical personnel. Hospital personnel who do not directly work with patients were categorized as non-clinical hospital staff, a group that included janitors, clerks, ambulance drivers, and firefighters.

#### Transmission tree of MERS hospital cluster in South Korea, 2015

We constructed the transmission tree of the South Korean outbreak, comprising 186 cases with the last case reported on 5 July 2015. For this purpose, we employed publicly available detailed case data from the WHO, the Korean Centers for Disease Control, and the Ministry of Health & Welfare of South Korea [13, 14, 33, 34]. The index patient developed symptoms on 11 May 2015 but was not diagnosed with MERS until 20 May 2015. This nosocomial outbreak involved 15 healthcare settings. While exposure information was available for all cases, only 168 (91 %) out of 185 transmission links tied to healthcare settings were ascertained through outbreak investigations.

#### Transmission tree of MERS hospital cluster in Al-Hasa, Saudi Arabia, 2013

We obtained a transmission tree for a nosocomial MERS outbreak comprising 25 cases that occurred between 1 April 2013 and 23 May 2013 in Al-Hasa, Saudi Arabia [11]. This nosocomial outbreak involved four healthcare facilities [11]. Transmission links were inferred for all secondary cases comprising this outbreak except for one case.

#### Transmission tree of SARS hospital cluster in Singapore, 2003

We obtained the transmission tree for a nosocomial SARS outbreak comprising 188 cases in Singapore between 25 February 2003 and 11 May 2003 [16]. The index patient was a local resident who developed symptoms in late February 2003 and was subsequently admitted to a hospital after returning from a holiday in Hong Kong. This nosocomial outbreak involved three major hospitals in Singapore [16].

#### Transmission tree of SARS hospital cluster in Toronto, Canada 2003

Detailed information was available on a nosocomial SARS outbreak in Toronto, Canada [17], resulting in 90 cases between 23 February 2003 and 15 April 2003 [17]. The index patient was a traveler returning from Hong Kong on 23 February 2003. This nosocomial outbreak developed in a single 249-bed secondary case community hospital [17]. Infection control precautions were implemented throughout the hospital including the closing of the hospital to admissions, closing of the outpatient clinics, and quarantine orders to discharged patients [17].

#### Ethics

MERS case data from Saudi Arabia were publicly available from the KSA Ministry of Health [2]. Similarly the dataset of MERS cases in South Korea was publicly available from the WHO, the Korean Centers for Disease Control, and the Ministry of Health & Welfare of South Korea [13, 14, 33, 34]. All of the data were de-identified. These openly available datasets were generated as part of emerging outbreak investigations and were, therefore, deemed exempt from institutional review board assessment.

### Analytical approach

We tabulated the pathogen-specific frequency of healthcare and familial exposure using individual-level data and aggregated case counts. To evaluate differences in exposure, we used chi-square and Fisher’s exact tests of independence in a cross-tabulation of exposure category and outbreak. Transmission trees allowed for tabulation of exposure frequency and number of secondary infections by pathogen, individual outbreak (n = 4), and disease generation—the time interval elapsed between successive generations of cases. Next, to quantify and compare the transmission potential and extent of transmission heterogeneity for MERS and SARS nosocomial outbreaks, we used an approach recently developed to characterize the distribution of cluster sizes for subcritical pathogens [35]. A hallmark of high transmission heterogeneity is a preponderance of very small and very large clusters (the latter being associated with super-spreading events), together with a low frequency of intermediate-size clusters. Based on this approach, we fit a negative binomial to the distribution of secondary cases obtained from the MERS and SARS transmission trees and estimated the reproduction number R and dispersion parameter k (with lower values indicating higher heterogeneity) [35]. Armed with these estimates, we simulated the expected distribution of future outbreaks that may occur in South Korea or elsewhere in terms of final size, peak size, and outbreak duration. We used branching process models [35] to simulate 5,000 MERS-like and 5,000 SARS-like outbreaks based on the distribution of secondary cases inferred from empirical transmission trees. Each simulated outbreak was initiated with a single infectious individual.

## Results

We analyzed the frequency of cases among healthcare workers for 973 MERS and 7,634 SARS patients (Table 1). The proportion of MERS cases among healthcare workers was similar in Saudi Arabia and South Korea (13.4 % versus 13.5 %). The proportion of healthcare workers among SARS cases varied from 19 % in China to 57 % in Vietnam, and was higher than that for MERS. The largest MERS outbreaks reported thus far have been greatly amplified in the healthcare setting, with the fraction of cases linked to hospitals ranging from 43.5 % for the 2014 outbreak in Jeddah, Saudi Arabia, to 100 % for both the 2013 outbreak in Al-Hasa, Saudi Arabia and the ongoing outbreak in South Korea (Table 2). The SARS outbreaks in Singapore, Toronto, China, and Vietnam were also primarily linked to healthcare settings with the proportion of cases tied to hospitals ranging from 73.5 % in Singapore to 100 % in Toronto (Table 2).

**Table 1:** Country-specific total number of cases and cases among healthcare workers for outbreaks of Middle East respiratory syndrome (MERS) [2, 13, 14, 33, 34] and severe acute respiratory syndrome (SARS) [29]

Coronavirus	Time period	Country	Total cases	Healthcare workers (%)
MERS	20 May–5 Jul 2015	South Korea	186	25 (13.4)
MERS	Jan 2013–May 2015	Saudi Arabia	787	106 (13.5)
SARS	23 Feb–12 Jun 2003	Canada	251	109 (43.4)
SARS	16 Nov–3 Jun 2003	China	5,327	1,002 (18.8)
SARS	15 Feb–31 May 2003	Hong Kong	1,755	386 (22.0)
SARS	25 Feb–5 May 2003	Singapore	238	97 (40.8)
SARS	23 Feb–14 Apr 2003	Vietnam	63	36 (57.1)

Information based on publicly available nationally aggregated case counts and patient-level data

**Table 2:** Total cases, cases linked to the healthcare setting, and cases among healthcare workers for individual nosocomial outbreaks of Middle East respiratory syndrome (MERS) and severe acute respiratory syndrome (SARS)

Coronavirus	Country	Time period	Total cases	Cases linked to healthcare settings (%)	Healthcare workers (%)	Sources
MERS	South Korea	20 May–5 Jul 2015	186	186 (100)	25 (13.5)	[13, 14, 33, 34]
	Al-Hasa, Saudi Arabia	1 Apr–23 May 2013	24	24 (100)	2 (8.3)	[11]
	Jeddah, Saudi Arabia	1 Jan–16 May 2014	225	98 (44)	78 (35.0)	[10]
Total MERS nosocomial outbreaks			435	308 (70.8)	105 (24.1)	
SARS	Singapore	25 Feb–11 May 2003	238	175 (74)	97 (41.0)	[16]
	Toronto, Canada	23 Feb–15 Apr 2003	216	216 (100)	92 (42.6)	[17, 51]
	Beijing, China	18 Mar–23 Apr 2003	125	103 (82)	67 (54.0)	[52]
	Vietnam	26 Feb–28 Apr 2003	63	52 (83)	37 (59.0)	[53]
	Hong Kong	15 Feb–31 May 2003	1,755	866 (49.3)	405 (23.1)	[54]
	Taiwan	Mar–Jun 2003	668	370 (55.4)	120 (18.0)	[37]
Total SARS nosocomial outbreaks			3,065	1782 (58.1)	818 (26.7)	

Information was obtained based on a literature search of hospital outbreaks of SARS and MERS

**Fig. 1:**
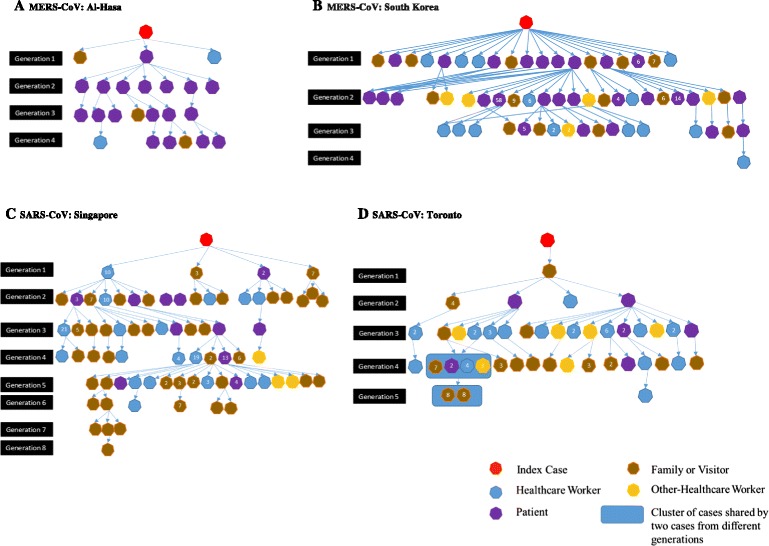
Transmission trees of Middle East respiratory syndrome (*MERS*) and severe acute respiratory syndrome (*SARS*) outbreaks linked to health-care settings. **a** MERS outbreak in Al-Hasa, Saudi Arabia, from 1 April to 23 May 2013 [11]. **b** MERS outbreak in South Korea from 20 May to 5 July 2015 [13, 33]. **c** Nosocomial SARS outbreak in Singapore from 25 February to 11 May 2003 [16]. **d** Nosocomial SARS outbreak in Toronto from 23 February to 5 April 2003 [17]. *Numbers* inside the nodes of the tree are used to indicate a group of cases rather than a single case. *Colors* are used to distinguish the index case from secondary cases and highlight different exposure categories among secondary cases, including patient, visitor or family member, healthcare worker, and non-clinical staff working in the hospital

**Fig. 2:**
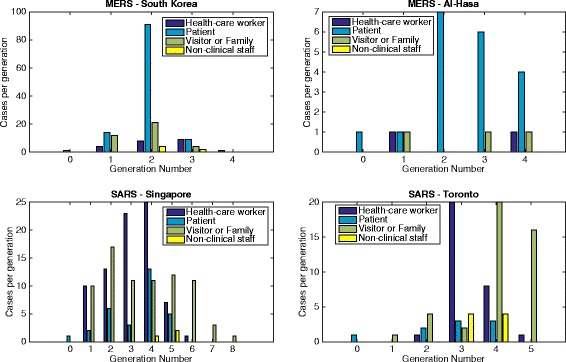
Total number of cases per disease generation and categorized according to exposure category (health-care worker, patient, visitor or family member, and non-clinical healthcare staff) for large nosocomial outbreaks of Middle East respiratory syndrome (*MERS*) and severe acute respiratory syndrome (*SARS*). Incidence starts at generation 0, which refers to the index case

**Fig. 3:**
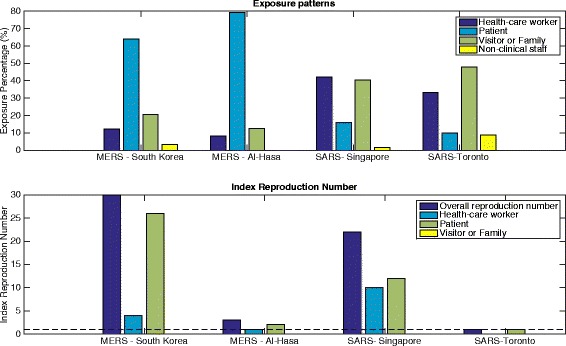
Exposure patterns among secondary cases (health-care worker, patients, visitor or family member, and non-clinical staff) throughout the entire outbreak (*top panel*) and for the first disease generation (*bottom panel*) for each of the Middle East respiratory syndrome (*MERS*) and severe acute respiratory syndrome (*SARS*) outbreaks linked to health-care settings. Bottom panel shows the total index reproduction number (R_0_) and a breakdown by exposure category of secondary case. The *horizontal dashed line* in the bottom panel at R = 1.0 is shown for reference

The transmission trees for the nosocomial MERS and SARS outbreaks are shown in Fig. 1 while the case progression by disease generation and exposure category is shown in Fig. 2. These outbreaks comprised only a few generations of infections, ranging from three to eight if the index case is considered to be generation 0. For MERS, the exposure patterns did not differ between the outbreaks (chi-square test; *P* = 0.36; Fig. 3), with the great majority of cases being patients (62.3–79.0 %) followed by family members (13–21 %). In contrast, SARS affected a larger proportion of healthcare workers (33–42 %) and family members (22–39 %) compared to the MERS outbreaks (chi-square test; *P* < 0.001; Fig. 3). There was no significant difference in relative exposure between the two SARS outbreaks (chi-square test; *P* = 0.2).

Of particular concern is the recent MERS outbreak in South Korea, which comprises a total of 186 cases including 25 healthcare workers, 116 patients (including the index patient), 39 visitors or family members, and 6 non-clinical staff cases (Fig. 1). A total of 30 secondary cases have been linked to the index patient in the first generation of the disease, 124 secondary cases have been reported for the second generation, 24 cases have been identified for the third generation, and one case has been reported for the fourth generation. This leads to a rough empirical estimate of the reproduction number according to disease generation of 30 for the first generation, 4.1 for the second generation, 0.2 for the third generation, and 0.04 for the fourth generation.

**Fig. 4:**
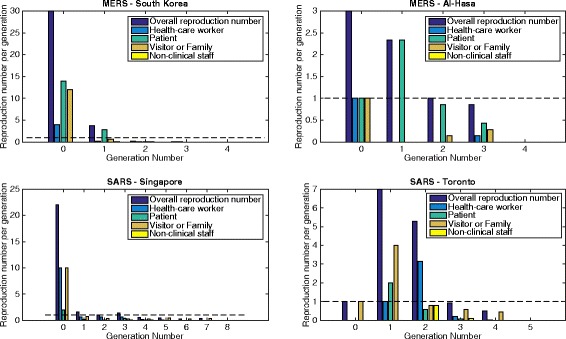
The reproduction number according to disease generations, R_g_ , starting with the index reproduction number (R_0_) at generation 0 and according to exposure category (healthcare worker, patients, visitors or family member, and non-clinical staff) for Middle East respiratory syndrome (*MERS*) and severe acute respiratory syndrome (*SARS*) outbreaks linked to healthcare settings. R_g_ denotes the mean number of secondary cases stemming from cases in generation g. The *horizontal dashed line* at R = 1.0 is shown for reference

The distribution of the individual reproduction numbers varied across individual outbreaks (Kruskal–Wallis test; *P* < 0.0001), and between MERS and SARS (Wilcoxon test; *P* < 0.0001). The reproduction number for the index case (generation 0) was high for three of the four outbreaks (range 1–30), including the ongoing outbreak in South Korea (Fig. 3). The reproduction number of the first two generations of secondary cases varied significantly for both SARS (median = 2, interquartile range 0.43–2.4) and MERS (range 0–80). For MERS, the reproduction number dropped below 1.0 in generation 2 for the outbreak in South Korea and generation 3 for the outbreak in Al-Hasa, whereas it did not drop below 1.0 until generation 4 for SARS (Fig. 4). Importantly, for MERS, a large proportion of the secondary cases were among patients visiting the same hospital facilities as the index patient in South Korea (69 %) and Al-Hasa, Saudi Arabia (100 %). Exposure categories were more balanced for SARS, with 47–57 % of secondary cases among healthcare workers, 24–30 % among visitors and family members, and 14–19 % among patients (Fig. 3).

Based on the transmission trees, one can identify several early super-spreading events fueling these outbreaks. The MERS outbreak in South Korea was characterized by three such events, the index patient who infected 30 secondary cases, and two patient cases of the second generation who infected 80 and 23 secondary cases each. In the MERS outbreak in Al-Hasa, Saudi Arabia, one patient infected seven other patients in the same hospital. The SARS outbreak in Singapore was associated with six super-spreading events of at least seven secondary cases each, whereas the SARS outbreak in Toronto was characterized by four super-spreading events of at least seven secondary cases each.

Next, we quantified transmission heterogeneity in these nosocomial outbreaks through the dispersion parameter k, which in turn allowed us to predict the size of future outbreaks of MERS (or SARS, should it reappear). By fitting a negative binomial distribution to the number of secondary cases in empirical transmission trees of realized outbreaks, we derived estimates of the mean reproduction number and the dispersion parameter (Table 3). The reproduction number indicates the average number of secondary cases per index case, while the dispersion parameter quantifies the degree of heterogeneity in the distribution of secondary cases. A lower value of k indicates more pronounced heterogeneity. Both MERS and SARS had reproduction numbers close to 1.0 in the hospital setting, although the estimate for MERS had broad confidence intervals, presumably due to smaller sample size. We found the dispersion parameter for MERS to be significantly lower than that for SARS, indicating higher heterogeneity in the distribution of secondary cases for nosocomial MERS outbreaks compared to SARS, and, in turn, a higher probability of super-spreading events of higher magnitude for MERS.

**Table 3:** Estimates of the reproduction number and the dispersion parameter k of Middle Eastern respiratory syndrome (MERS) and severe acute respiratory syndrome (SARS) in the hospital

Coronavirus	Mean R (95 % CI)	k (95 % CI)
MERS	0.91 (0.36, 1.44)	0.06 (0.03, 0.09)
SARS	0.95 (0.67, 1.23)	0.20 (0.13, 0.27)

**Fig. 5:**
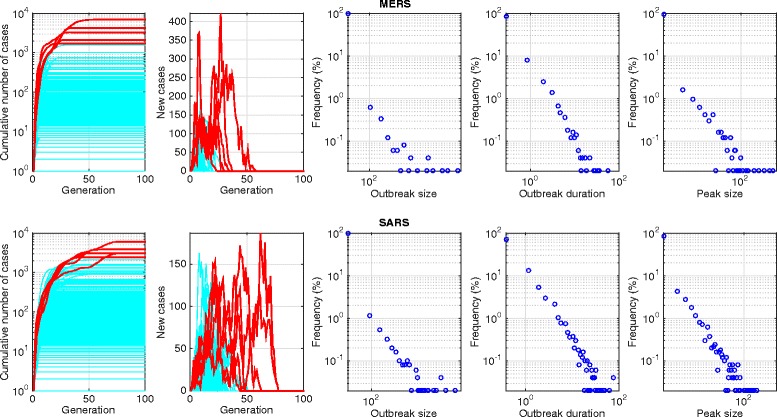
Outbreak simulations for (*top*) Middle Eastern respiratory syndrome (*MERS*) and (*bottom*) severe acute respiratory syndrome (*SARS*) through branching process models using parameters shown in Table 3. Curves are derived from 5,000 outbreak realizations. The distributions of outbreak size, duration, and peak size are well characterized by power-law-like distributions. The cumulative and generation-based incidence curves for the five largest outbreaks are highlighted in *red*; while smaller outbreaks are displayed in cyan. *Right panels* illustrate the expected frequency of occurrence of an outbreak of a given size

**Fig. 6:**
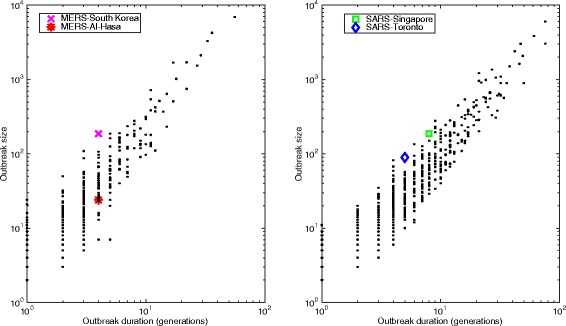
Scatter plot of expected outbreak size and duration for 5,000 simulated outbreaks derived via branching process models of Middle Eastern respiratory syndrome (*MERS*) and severe acute respiratory distress (*SARS*) using parameters shown in Table 3. Data points for observed MERS and SARS outbreaks are shown in color for reference

Outbreak simulations for both MERS and SARS showed marked variability in outbreak characteristics, with the distribution of outbreak size, peak size, and outbreak duration following power-laws (Fig. 5). Figure 6 displays the scope of outbreak size and duration for MERS and SARS directly derived from the joint probability distribution of outbreak outcomes. For illustration purposes, the probability of a future outbreak greater than 100 cases is 1.2 % for MERS and 2.3 % for SARS. The higher transmission heterogeneity of MERS manifests itself by a more skewed distribution of outbreak sizes and sharper peaks expected during the largest outbreaks, relative to SARS (Fig. 5).

While the great majority of outbreak simulations only comprised a few cases (Fig. 5), a few outbreak simulations for both MERS and SARS demonstrate the potential for multi-modal outbreaks characterized by larger total size and duration that result from low-probability super-spreading events (Additional file 1: Figs. S1 and S2). Moreover, these simulations are consistent with observed multi-modal outbreak curves for SARS and MERS as shown in Additional file 1: Fig. S3. Overall, multi-modal outbreaks of MERS tend to have higher peak size but shorter duration.

## Discussion

This is the first head-to-head comparison of exposure and transmission patterns of large hospital clusters of MERS and SARS, with a focus on the recent May–July 2015 MERS outbreak in South Korea. Nosocomial outbreaks of both diseases were characterized by high transmission heterogeneity, with three to six super-spreading events identified during the early stages of transmission. Intriguingly, there was a systematic difference in the exposure patterns of MERS and SARS, with a majority of MERS cases occurring among patients who sought care in the same facilities as the index case, and a greater concentration of SARS cases among healthcare workers throughout the outbreak. Exposure patterns differed slightly by disease generation, however, especially for SARS. Although the number of hospital outbreaks available for study remains limited, comparison of the distribution of secondary cases suggests a similar transmission potential within the hospital for both viruses (R_0_ close to 1.0), but greater transmission heterogeneity for MERS than SARS. Our study suggests that the South Korean outbreak follows a similar progression to previously described hospital clusters involving coronaviruses. In these clusters, an early super-spreading event linked to a single hospitalized index case generated a disproportionate number of infections, while the transmission potential diminished greatly in subsequent generations, indicating a significant effect of active case detection and control interventions. Our simulations suggest that the probability of a future hospital outbreak of MERS larger than the South Korean 2015 outbreak (i.e., >186 cases) is only of the order of 1 %.

Super-spreading events tied to nosocomial outbreaks of MERS and SARS have been attributed in part to diagnostic delays, which increase the window of opportunity for generation of secondary cases in settings with suboptimal infection control measures [14, 16, 36, 37]. Accordingly, the index patient of the MERS outbreak in South Korea was diagnosed 9 days after the onset of symptoms, and generated an estimated 30 secondary cases [14]. Similarly, for the nosocomial SARS outbreak in Singapore, the index patient infected 22 secondary individuals and was isolated 5 days after admission to the hospital [38]. Furthermore, for a nosocomial SARS outbreak in Taiwan, the index case was diagnosed with SARS and admitted to the hospital 6 days after the onset of symptoms, infecting 137 secondary SARS cases including 45 healthcare workers [37]. Super-spreading events are the hallmark of a highly heterogeneous transmission pathway and are potentially mediated by individual variation in infectivity (through viral shedding) or in the number of contacts [23]. These characteristics are shared by several directly transmitted infectious diseases; comparison of the transmission heterogeneity parameter k suggests that nosocomial transmission of MERS could be more prone to super-spreading events than SARS, monkeypox, Ebola, or measles in the post-elimination era [23, 35, 39, 40]. Additional data on nosocomial MERS outbreaks would be needed, however, to confirm these findings, especially because heterogeneity may differ between outbreaks and countries, and decrease after interventions are put in place [23].

The reproduction number for secondary cases during transmission chains of MERS in the Middle East has been estimated to lie below the epidemic threshold at R = 1 [1, 5, 6], whereas past outbreaks of SARS have been characterized by an overall reproduction number between 2 and 3 before interventions were implemented [41, 42]. Based on the distribution of secondary cases in the Al-Hasa and South Korean outbreaks, we cannot rule out that the reproduction number of MERS is above 1 in the healthcare setting, although our confidence intervals are large. Our estimates, however, are substantially lower than estimates based on more recent nosocomial outbreak data from Saudi Arabia [43]. Moreover, a recent analysis of MERS cluster sizes reported up to 8 August 2013 [25] estimated a subcritical R at 0.6 and less heterogeneity than our study (k = 0.24), perhaps owing to the choice of a different time period, or reliance on cluster size data rather than the distribution of secondary cases during hospital outbreaks as in our study. As a result, the expected probability of large outbreaks is smaller in [25] than in our study (0.01 % for an outbreak larger than the realized South Korean one, versus ~1 % in our study). Similar findings were obtained by another study that analyzed the outcomes of 36 historical MERS importation events ,including the recent South Korean outbreak, in terms of outbreak size and total number of disease generations (R = 0.75 and k = 0.14) [26]. Differences between studies are in part driven by methodology, because reproduction number estimates could be inflated in studies that do not account for transmission heterogeneity. Further, each study focused on a different subset and time period of the MERS epidemic, and to our knowledge our study is the first to dissect transmission dynamics in the hospital setting. Of note, our approach did not explicitly integrate the effect of increased case detection, contact tracing, and infection control interventions, which likely helped stamp out the MERS and SARS outbreaks after a few disease generations. Overall, while transmission of MERS (and SARS) appears critical in the hospital setting before active case detection and interventions are in place, it is thought that the transmission potential of these coronaviruses remains subcritical in the community [6].

The ongoing South Korean cluster appears to be large in terms of initial super-spreading events, and we cannot rule out a different transmission potential of MERS in South Korea owing to particular climatological conditions, characteristics of the hospital system, and cultural factors, including the tendency of family members and visitors to be involved in the nursing work of hospitalized relatives [44]. Interestingly, we found that the relative exposure patterns differed between SARS and MERS, with a higher frequency of healthcare workers for SARS, and a predominance of patients visiting the same hospital as the index case for MERS. For both pathogens, family members came second in terms of risk category. These differences would be worth investigating further because they could signal an improvement in healthcare worker precautions since the SARS outbreak in 2003, different patient care habits in South Korea characterized by higher involvement of family members, or slightly different routes of transmission for these two coronaviruses.

Our findings derived from the comparative analysis of nosocomial outbreaks of MERS and SARS indirectly support the need for rapid case detection, enhanced and sustained infection control measures, and effective isolation and quarantine strategies in order to prevent or promptly control potential MERS and SARS outbreaks, which is in line with past modeling studies of the transmission dynamics of SARS in 2003 [41, 42, 45–48]. These measures include droplet precautions, e.g., wearing surgical masks, and contact precautions, e.g., wearing gown and gloves in the patients’ room [49]. In addition, the super-spreader events that are key to amplify nosocomial transmission of MERS and SARS outbreaks [23] support the adoption of airborne precautions that include at least six hourly air changes in treatment rooms [50]. A thorough investigation of the transmission pathways from a single index case to 30 secondary cases in healthcare settings in South Korea is needed.

Our study is not exempt of limitations. First, our data from South Korea may be prone to right censoring, as further transmission events stemming from individuals under quarantine or currently hospitalized individuals cannot be ruled out in the near future. However, the outbreak appears to be on an imminent path to extinction given that no new cases have been reported in South Korea since 4 July 2015. Second, we were not able to characterize risk of infection according to exposure category in absolute terms (e.g., healthcare workers, patients, visitors) because relevant denominator data were not available for secondary cases. For this reason, we focused our analysis on relative comparisons of exposure categories between individual outbreaks, disease generations, and virus types. Third, we focused on outbreaks with detailed transmission trees, and thus, contact-tracing activities must have been effectively carried out during those outbreaks, which may in turn have affected subsequent exposure and transmission patterns. Overall, most methods used to quantify transmission potential and transmission heterogeneity are prone to reporting biases, especially because larger clusters and more severely ill patients tend to be overly represented in any surveillance dataset [1, 6, 24]. However, large hospital outbreaks that persist for several generations are worth studying because they offer a useful window on the distribution of secondary cases, for a particularly important subset of the epidemic.

## Conclusion

We have carried out a first head-to-head comparison of exposure and transmission patterns for large hospital clusters of MERS and SARS, including the most recent May–July 2015 MERS outbreak in South Korea. Our findings confirm the importance of super-spreading events in the healthcare setting for the transmission dynamics of both coronaviruses, an effect that may be even more pronounced for MERS than SARS. As a result, large outbreaks of MERS, although rare, can happen and can generate very sharp incidence peaks that may be difficult to control. Differences in the relative exposure of the two viruses could signal changes in hospital healthcare practices over time and/or different mechanisms of transmission, which would be worth investigating further. Our data are consistent with the benefits of rapid case detection and strict adherence to infection control measures, which can rapidly reduce the risk of super-spreading events and therefore the size of the nosocomial outbreaks [10, 11, 16, 17]. More broadly, our study emphasizes the importance of individual patient data and transmission tree information to dissect the progression of subcritical outbreaks of key interest. Overall, the South Korean experience with MERS underscores the potential risk of importation of emerging infectious diseases into other regions of the world and the need to better understand the cross-species transmission mechanisms of MERS in the Arabian Peninsula [20]. The South Korean MERS outbreak is a wake-up call emphasizing the need for flexible epidemiological surveillance systems and strong public health infrastructure to quickly detect and stamp out potential outbreaks, including in countries with no prior MERS experience.

## Additional file

Additional file 1:
**Supplementary figures. Figure S1.** Outbreak simulations for MERS that demonstrate the potential for multi-modal outbreaks characterized by larger total size and duration that result from low-probability super-spreading events. **Figure S2.** Outbreak simulations for SARS that demonstrate the potential for multi-modal outbreaks characterized by larger total size and duration that result from low-probability super-spreading events. **Figure S3.** Time series of incident cases of MERS and SARS outbreaks display multi-modal epidemic patterns. (PDF 3765 kb)
