# Supplementary material for: Transmission characteristics of MERS and SARS in the healthcare setting: a comparative study
Source: BMC Med. 2015 Sep 3;13:210. doi: 10.1186/s12916-015-0450-0 (PMC4558759; doi:10.1186/s12916-015-0450-0)

Additional file 1: Supplementary figures

Figure S1. Outbreak simulations for MERS that demonstrate the potential for multi-modal outbreaks characterized by larger total size and duration that result from low-probability super-spreading events. Simulations were obtained through branching process models using parameters shown in Table 3.

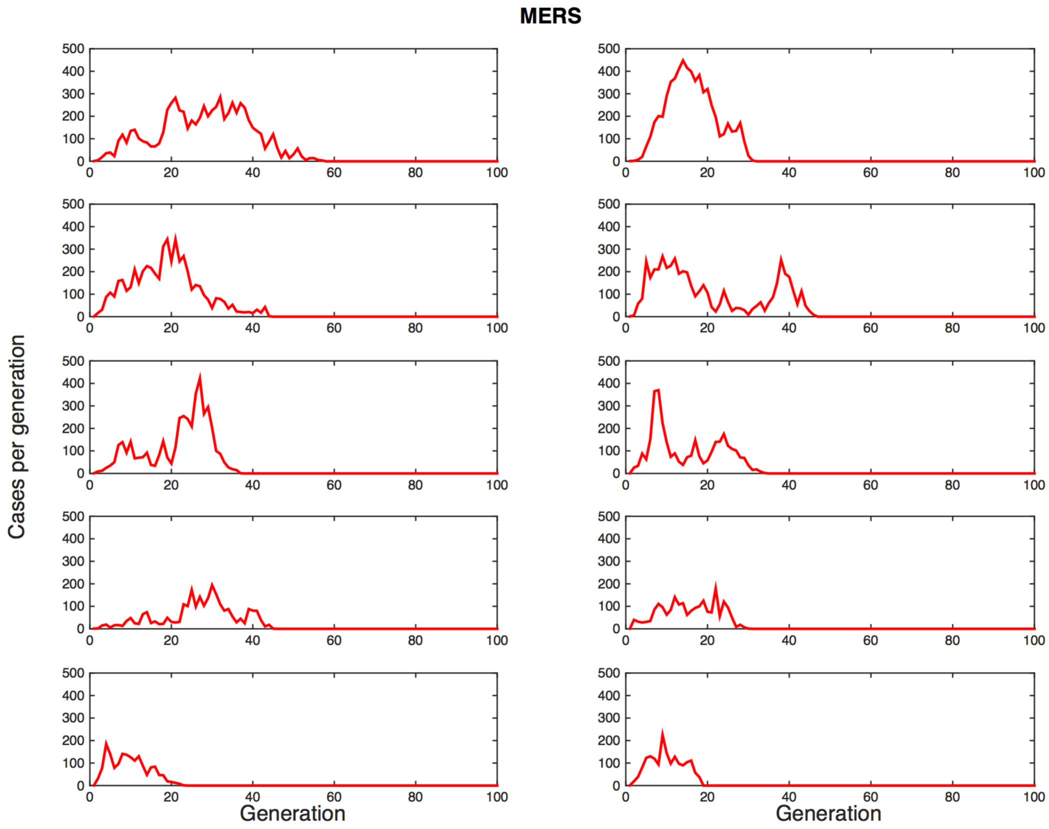

Figure S2. Outbreak simulations for SARS that demonstrate the potential for multi-modal outbreaks characterized by larger total size and duration that result from low-probability super-spreading events. Simulations were obtained through branching process models using parameters shown in Table 3.

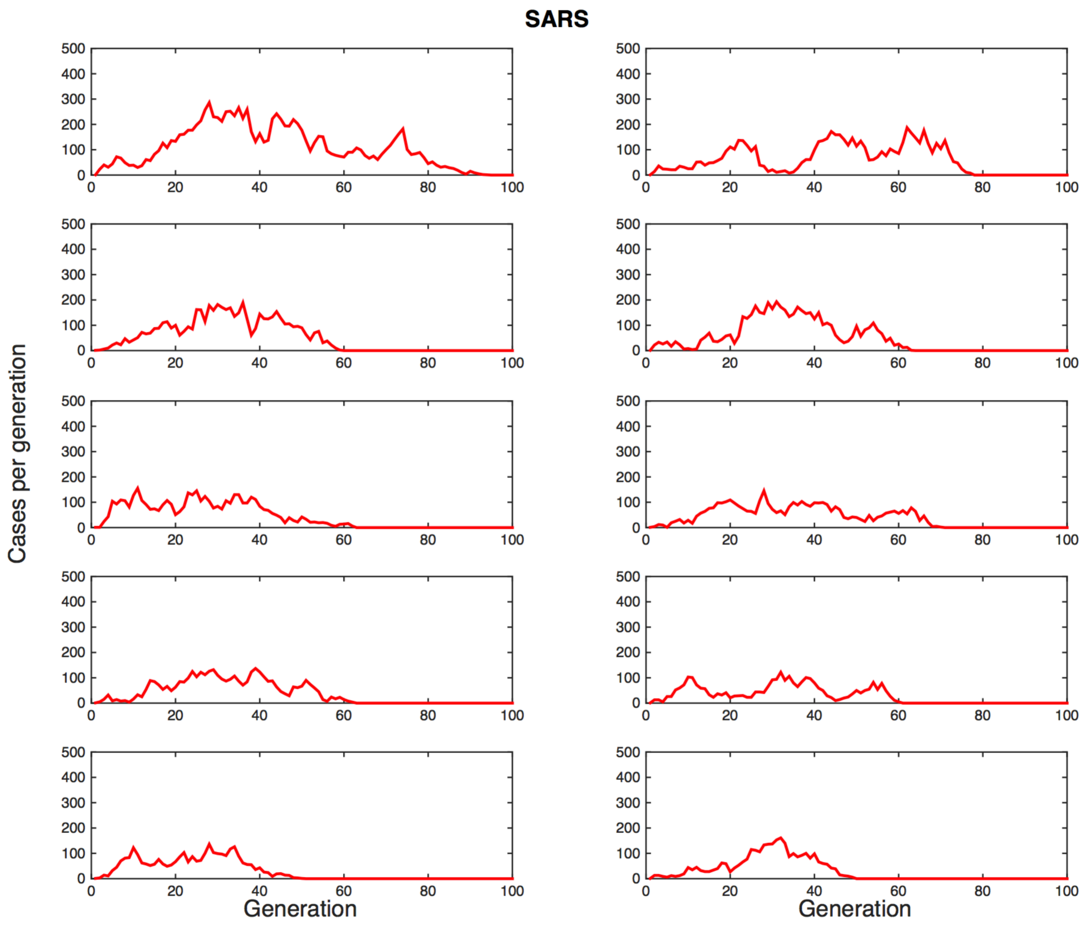

Figure S3. Time series of incident cases of MERS and SARS outbreaks display multi-modal epidemic patterns. SARS outbreaks in Singapore and Toronto occurred from 23 February 2003 to 8 June 2003. The MERS outbreak in South Korea occurred from 10 May to 5 July 2015 while the MERS outbreak in Al-Hasa, Saudi Arabia occurred from 8 April to 12 May 2015.

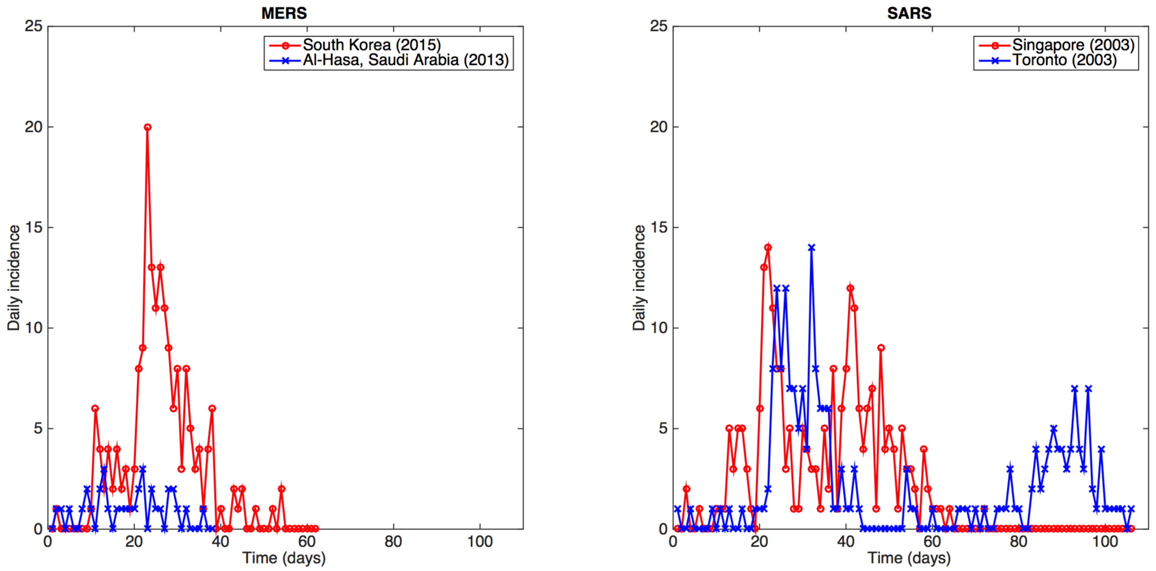

Supplement: Additional file 1: — Supplementary figures. Figure S1. Outbreak simulations for MERS that demonstrate the potential for multi-modal outbreaks characterized by larger total size and duration that result from low-probability super-spreading events. Figure S2. Outbreak simulations for SARS that demonstrate the potential for multi-modal outbreaks characterized by larger total size and duration that result from low-probability super-spreading events. Figure S3. Time series of incident cases of MERS and SARS outbreaks display multi-modal epidemic patterns. (PDF 3765 kb) [file 12916_2015_450_MOESM1_ESM.pdf]
